# A Mendelian randomization study on the association between systemic inflammatory regulators and essential and secondary hypertension

**DOI:** 10.3389/fcvm.2024.1335395

**Published:** 2024-10-10

**Authors:** Xiang Ji, Jiao Ren, Feng Dong, Wei Peng

**Affiliations:** ^1^ Department of General Medicine, Affiliated Hospital of Shandong University of Traditional Chinese Medicine, Jinan, Shandong Province, China; ^2^ Department of Psychosomatic Medicine, Affiliated Hospital of Shandong University of Traditional Chinese Medicine, Jinan, Shandong Province, China

**Keywords:** hypertension, inflammation, Mendelian randomization, TNFB, MIP1b

## Abstract

**Background:**

Inflammation is an important driver of hypertension with numerous components, and there is a paucity of research on the specific inflammatory factors that induce hypertension; therefore, we wanted to investigate the relationship between specific inflammatory factors and hypertension.

**Purpose:**

A two-sample Mendelian randomization (MR) study was conducted to assess the causal relationship between systemic inflammatory regulators and hypertension (primary or secondary types).

**Method:**

a large-scale, published genome-wide association study (GWAS) meta-analysis encompassing 41 cytokines (involved 8,293 Finnish participants from three independent population cohorts: the Cardiovascular Risk in Young Finns Study (YFS), FINRISK1997, and FINRISK2002.)were utilized, a variety of analyses including MR-Egger, weighted median, simple mode and weighted mode were used as sensitive analyses, to corroborate the causal relationship between inflammatory regulators and hypertension. Additionally, we used MR-Egger intercept test and Mendelian Randomization Pleiotropy RE Sidual Sum and Outlier (MR-PRESSO global test) to further evaluate the presence of horizontal pleiotropy.

**Results:**

3 inflammatory regulators were found related to secondary hypertension, TNFb was negatively associated with risk of secondary hypertension, with a OR of one SD increase in genetically predicted TNFb causing 16.6% (95% CI: 4.4%–27.1%) lower risk of secondary hypertension. Similar trend was also found in MIP1b (OR = 0.91; 95% CI 0.84–0.99, *p* = 0.024) and MIG (OR = 0.88; 95% CI 0.78–0.99, *p* = 0.040). Additionally, there was not any evidence of 41 inflammatory regulators associated with primary hypertension.

**Conclusion:**

This study supports a negative correlation between TNFb, MIP1b, MIG and secondary hypertension.

## Background

Hypertension is a common chronic disease causing cardiac and cerebrovascular complications, increasing deaths annually (1). Despite the availability of a wide range of antihypertensive medications, 71% of patients fail to control their blood pressure below 130/80 mmHg (2) (2023 ESH Arterial Hypertension Management Guidelines European Society of Hypertension Arterial Hypertension Management Working Group: Recognized by the International Society of Hypertension (ISH) and the European Kidney Association (ERA)—doi: 10.1097/HJH0000000003480). At present, the mechanisms underlying the development of hypertension are still not fully understood clinically. Although there are a large number of antihypertensive drugs that can act on this type of disease, the majority of patients are still not effectively controlled (3). This highlights the urgent need for further improvement in risk assessment. Despite recent significant progress, identifying and managing primary and secondary hypertension still faces significant challenges.Therefore, there is an urgent clinical need for treatments and drugs with better efficacy.

Hypertension is the result of a combination of environmental and genetic factors, an important one being low-grade inflammation (4). Hypertension is caused by the activation of complement (inflammasome) and changes in the phenotype of circulating immune cells, especially bone marrow cells. These inflammatory events are interdependent and ultimately participate in the progression of hypertension through mechanisms involving oxidative stress, endogenous protein modifications, and alterations in antigen processing and presentation (5). Microvascular remodeling, autonomic nervous system (ANS) imbalance, and RAAS activation are the main pathological mechanisms of hypertension (6). Inflammation has been found to be associated with endothelial cell activation and dysfunction in hypertension, and the two pathologic processes are interrelated. Clinical observation evidence and animal experiments both indicate that inflammation plays an important role in the development of hypertension (7). The biomarkers of inflammation, including high sensitivity C-reactive protein, various cytokines, and products of complement pathways, are elevated in hypertensive patients (8). The inflammation-mediated endothelial cell activation is an important step in triggering hypertension. Both innate immunity and adaptive immunity can promote blood pressure elevation by triggering vascular inflammation and microvascular remodeling. For example, classically activated macrophages (M1), neutrophils, and dendritic cells cause hypertension by secreting inflammatory cytokines. Interferon gamma (IFN - *γ*) and interleukin-17 (IL-17) induce oxidative stress damage and endothelial dysfunction, leading to hypertension (6). Long term inflammatory processes can increase the production of ROS, leading to oxidative stress and endothelial dysfunction, regulating the tension and structure of blood vessels (9). It can provide a substrate for the inflammation of primary hypertension (2). While, hypertension also deteriorates the endothelium, which leads to further inflammation, deteriorating blood pressure in a vicious cycle. Hypertension is proved accompanied by the activation of inflammasome complement and circulating immune cells (8). It is suggested that inflammatory factors are involved in HP progression or are associated with patient mortality, but the exact correlation is unknown.

Mendelian randomization (MR) is an analytical method that uses genetic variation in non-experimental data to infer causal effects of exposure on outcomes (10). Because alleles are randomly assigned during meiosis, MR reduces traditional confounding variables and reverse causation, providing better evidence for causal inference (11). MR analysis of two samples allows researchers to assess instrument-exposure and instrument-outcome associations in two independent population samples, thereby enhancing the applicability and validity of the test (12). Therefore, we aimed to utilize serum data from Finnish patients with hypertension and the International Consortium on Blood Pressure (ICBP) Genome-Wide Associated Study (GWAS) population to perform Mendelian randomization (MR) analyses, in order to further explore the correlation between specific 43 inflammatory cytokines and hypertension progression, and then to further explore risk factors for hypertension stratification.

## Material and methods

### Study design

A two-sample Mendelian randomization (MR) study was conducted to assess the causal relationship between systemic inflammatory regulators and hypertension (primary or secondary types). All data used in this study were obtained from previously published research, with informed consent already acquired in the original studies. We first employed genetic variants associated with inflammatory regulators to determine their causal relationship with hypertension. In our study, the chosen valid instrumental variables (SNPs) strictly adhered to the three assumptions of Mendelian randomization analysis.

### Data source for systemic inflammatory regulators

In this study, we utilized a large-scale, published genome-wide association study (GWAS) meta-analysis encompassing 41 cytokines, representing the most comprehensive data set available to date (PMID: 27989323). The study involved 8,293 Finnish participants from three independent population cohorts: the Cardiovascular Risk in Young Finns Study (YFS), FINRISK1997, and FINRISK2002. Cytokines were measured in EDTA plasma from FINRISK1997, heparin plasma from FINRISK2002, and serum from YFS. Subjects with cytokine concentrations below or above the detection limits of laboratory analysis were omitted from the analyses. Additionally, cytokines with more than 90% missing values (7 out of 48) were excluded from the study. Generally, the YFS participants are generally younger than those in the FINRISK1997 and FINRISK2002 studies, with an average age of 37 compared to 60 years. More detailed characteristics of above three study cohorts are reported in Supplementary Table S1.

### Data sources for hypertension

GWAS summary data for primary or secondary hypertension were both obtained from FinnGen consortium R9 release data. The FinnGen study aims to produce genomic data with linkage to national health register data of 500,000 Finnish individuals, which is enriched for disease end points (PMID: 36653562). The end point “Hypertension, essential” and “Secondary hypertension” were used in this study. These two GWAS consisted of 92,462 cases and 265,626 controls and 2,597 cases and 191,924 controls, respectively, and the median age at first event was 65 years for “Hypertension, essential” and 57 years for “Secondary hypertension”. The following variables were adjusted during the analysis: gender, age, first 10 main components, and genotyping batch. More details about the endpoint were provided at https://risteys.finregistry.fi/.

### Selection of instrumental variables

Given a *p*-value threshold of <5 × 10^−8^ for SNP selection did not yield three or more significant SNPs for all 41 systemic inflammatory regulators, to obtain a more comprehensive set of SNPs for inflammatory regulators, we adjusted the significance threshold to *P* < 5 × 10^−6^. We employed a stringent clumping procedure to ensure that independent SNPs met the linkage disequilibrium (LD) coefficient criteria (*r*^2^ < 0.001, window size = 10,000 kb) and removed palindromic SNPs. To mitigate the impact of weak instrumental variables on the MR model's performance, we used the *F* statistic of SNPs to evaluate the strength of instrumental variables. Typically, an *F* value >10 indicates no apparent bias caused by weak IVs.

### Statistical analysis

Given it provides the highest statistical power, the inverse-variance weighted (IVW) approach was used as our primary analysis (PMID: 26661904). When these conditions are not met, the results may be biased. Therefore, a variety of analyses including MR-Egger, weighted median, simple mode and weighted mode were used as sensitive analyses, to corroborate the causal relationship between inflammatory regulators and hypertension (PMID: 29040600). Additionally, we used MR-Egger intercept test and Mendelian Randomization Pleiotropy RESidual Sum and Outlier (MR-PRESSO global test) to further evaluate the presence of horizontal pleiotropy (PMID: 29686387, PMID: 26050253). An MR-Egger regression intercept not equal to zero and *P* < 0.05 indicates that the inflammatory factors may not be the sole pathway through which IVs influence hypertension. The MR-PRESSO global test can further correct for potential outlier pleiotropy by removing outlier SNPs when necessary. We also performed heterogeneity tests on IVW and MR-Egger results using the Cochrane *Q* test; when *P* < 0.05, heterogeneity is present, and we need to evaluate outliers in conjunction with other results. It is generally believed that the heterogeneity of a single result will not significantly impact the prediction of causal relationships. The leave-one-out sensitivity analysis, which involves the sequential exclusion of SNPs, is commonly used to find and eliminate SNPs that have a significant impact on the results, ensuring the reliability of the outcomes (PMID: 27749700).

We consider a significant causal relationship to exist between inflammatory factors and hypertension when the following three conditions are met: (1) A significant difference exists in the primary analysis method IVW results (*P* < 0.05); (2) Other sensitive methods are consistent with the IVW estimate direction; (3) No pleiotropy was found in MR-Egger intercept test or MR-PRESSO global test results (*P* < 0.05).

## Results

### Selection of instrumental variables (IVs)

A total of 840 SNPs were screened as IVs for 41 systemic inflammatory regulators. These IVs explained 2.3%–24.7% of the variance and the F-statistics of them were all greater than 10 (ranged 20.1–128.3), signifying the robustness and strength of the IVs employed in this study. This indicates the absence of any significant weak instrument bias in the results, making the findings reliable and acceptable (Supplementary Table S2).

### Two-sample MR analysis

Among all 41 inflammatory regulators analyzed in our study, 3 inflammatory regulators were found related to secondary hypertension according to the criteria mentioned above in which the IVW method demonstrated a significant difference (*P* < 0.05), and the other methods indicated consistent directions. In the IVW analysis, genetically predicted TNFb was negatively associated with risk of secondary hypertension, with a OR of one SD increase in genetically predicted TNFb causing 16.6% (95% CI: 4.4%–27.1%) lower risk of secondary hypertension. Similar trend was also found in MIP1b (OR = 0.91; 95% CI 0.84–0.99, *p* = 0.024) and MIG (OR = 0.88; 95% CI 0.78–0.99, *p* = 0.040) (Table 1, Supplementary Table S3). Additionally, there was not any evidence of 41 inflammatory regulators associated with primary hypertension (Supplementary Table S3).

**Table 1:** Result of MR estimates for three systemic inflammatory regulators.

Exposure	Outcome	MR method	No. of SNP	OR(95% CI)	*P*-value
TNFb	Secondary hypertension	MR Egger	4	0.85 (0.68–1.06)	0.29
TNFb	Secondary hypertension	Weighted median	4	0.86 (0.72–1.01)	0.06
TNFb	Secondary hypertension	Inverse variance weighted	4	0.84 (0.73–0.96)	0.0095
TNFb	Secondary hypertension	Simple mode	4	0.91 (0.72–1.13)	0.45
TNFb	Secondary hypertension	Weighted mode	4	0.84 (0.70–1.02)	0.17
MIP1b	Secondary hypertension	MR Egger	18	0.89 (0.79–1.01)	0.1
MIP1b	Secondary hypertension	Weighted median	18	0.87 (0.77–0.98)	0.018
MIP1b	Secondary hypertension	Inverse variance weighted	18	0.91 (0.84–0.99)	0.024
MIP1b	Secondary hypertension	Simple mode	18	0.91 (0.73–1.13)	0.41
MIP1b	Secondary hypertension	Weighted mode	18	0.90 (0.81–0.99)	0.05
MIG	Secondary hypertension	MR Egger	14	0.76 (0.57–1.00)	0.07
MIG	Secondary hypertension	Weighted median	14	0.88 (0.74–1.04)	0.13
MIG	Secondary hypertension	Inverse variance weighted	14	0.88 (0.78–0.99)	0.04
MIG	Secondary hypertension	Simple mode	14	0.85 (0.65–1.11)	0.25
MIG	Secondary hypertension	Weighted mode	14	0.87 (0.68–1.11)	0.28

### Sensitivity analyses and leave-one-out analysis

**Figure 1:**
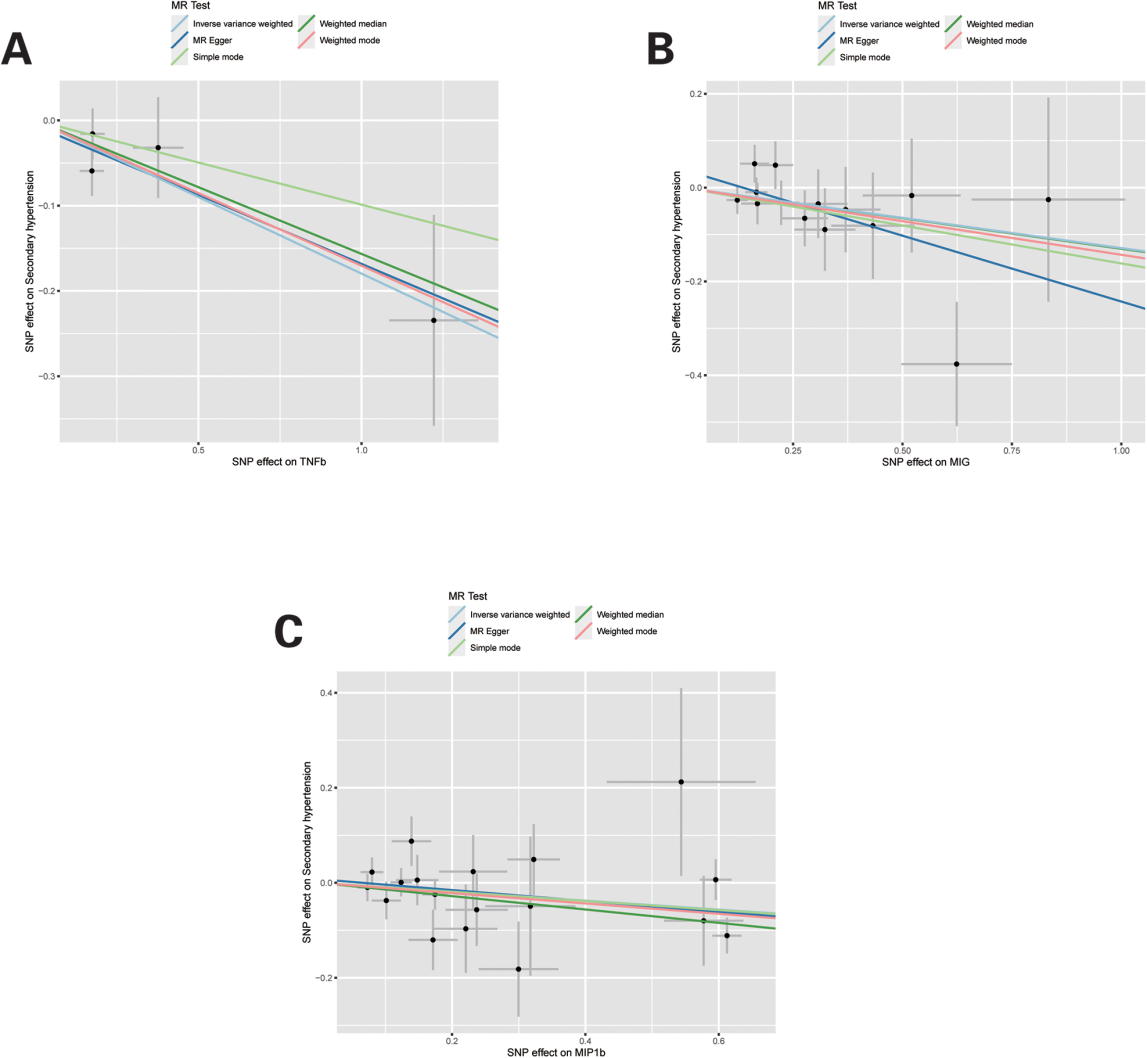
Scatter plots of Mendelian randomization (MR) analyses for TNFb, MIG and MIP1b in secondary hypertension. Individual inverse variance (IV) associations with systemic inflammatory regulators risk are displayed vs. individual IV associations with Secondary hypertension in black dots. The 95% CI of odd ratio for each IV is shown by vertical and horizontal lines. The slope of the lines represents the estimated causal effect of the MR methods.

**Figure 2:**
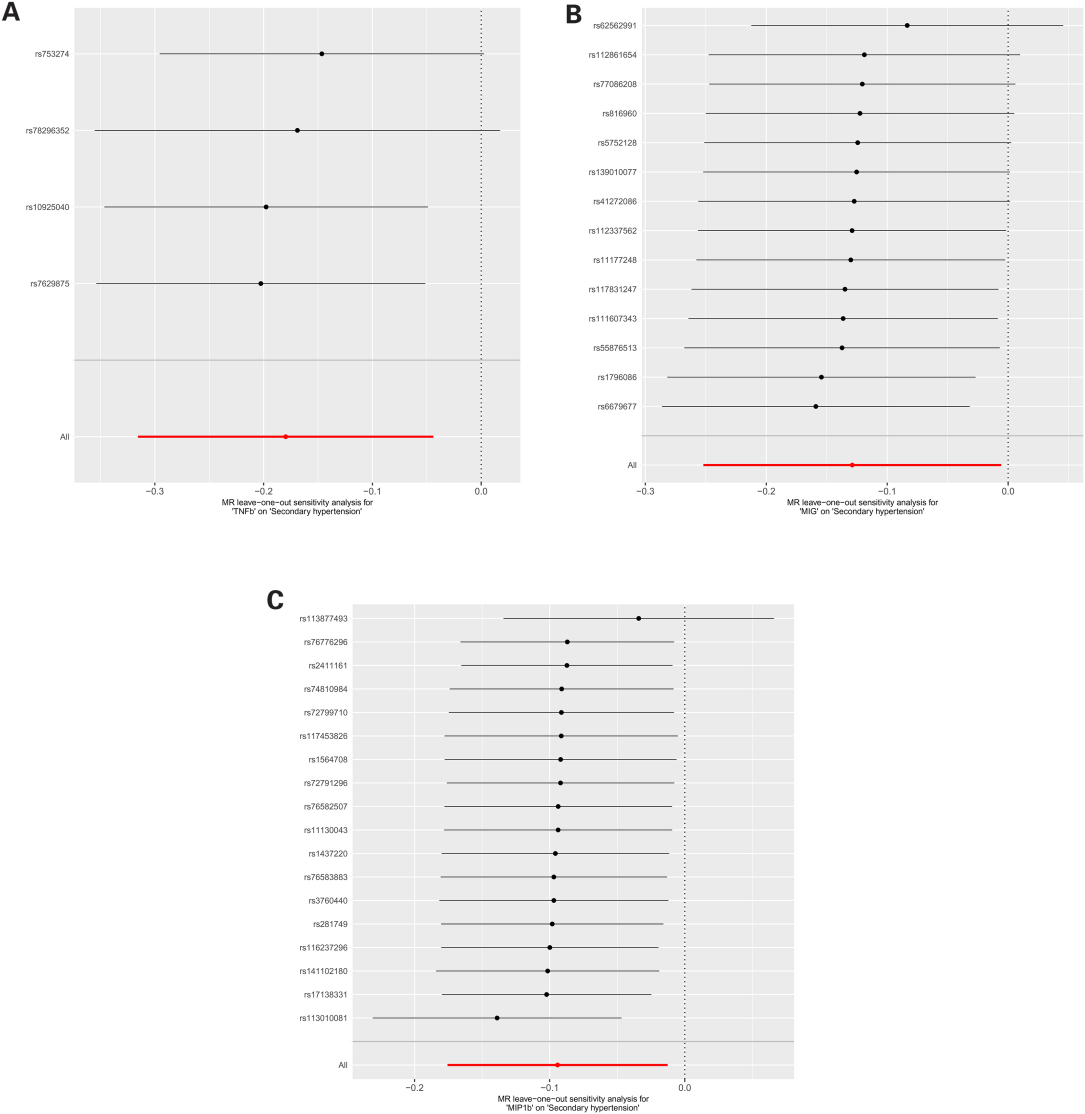
**(A)** MR leave-one-out sensitivity analysis for “TNFb” on “Secondary hypertension”. **(B)** MR leave-one-out sensitivity analysis for “MIG” on “Secondary hypertension”. **(C)** MR leave-one-out sensitivity analysis for “MIP1b” on “Secondary hypertension”.

For these three inflammatory regulators, there was not any heterogeneity found in the IVW method or the MR-Egger regression, the *P*-value for Cochran's *Q* test were all >0.05 (Supplementary Table S4). MR Egger regression intercepts were near zero and revealed little evidence of horizontal pleiotropy in the IVs (Supplementary Table S5). Using the MR-PRESSO global outlier test, no evidence for outliers was observed (Supplementary Table S5). The scatter plots did not demonstrate any potential outlier in the IVs for these three inflammatory regulators (Figure 1). In the leave-one SNP-out analyses, the risk estimates of all inflammatory regulators generally remained consistent after eliminating each single SNP at a time, that it is, not any leverage point with high influence was identified (Figure 2). The full results of MR estimate for the 41 inflammatory regulators on hypertension are presented in Supplementary Tables S3–S6.

## Discussion

Numerous studies based on the inflammatory response in hypertension have made breakthroughs and confirmed the efficacy of many drugs on the inflammatory response in Primary and secondary hypertension (13). 5%–10% of hypertensive patients suffer from secondary hypertension. The main causes of secondary hypertension are substantial kidney disease, primary aldosteronism, and renal vascular hypertension. Identifying these patients can enable etiological management of potential diseases (14). Many inflammatory markers have been shown to be elevated in hypertensive patients, such as CRP, the most widely studied inflammatory marker, which has been shown to have elevated levels in the blood of hypertensive patients and has been associated with prognosis. Elevated levels of other markers have also been observed in hypertensive patients, such as IL-1β, IL-6, TNF-α, and leukocytes (15). However, there are currently no reports on distinguishing inflammatory factors from primary and secondary hypertension. In this study we utilized a large-scale, published genome-wide association study (GWAS) meta-analysis encompassing 41 cytokines (involved 8,293 Finnish participants from three independent population cohorts: the Cardiovascular Risk in Young Finns Study (YFS), FINRISK1997, and FINRISK2002.), to corroborate the causal relationship between inflammatory regulators and hypertension. Finally, we found 3 inflammatory regulators were found related to secondary hypertension according to the criteria mentioned above in which the IVW method demonstrated a significant difference (*P* < 0.05). TNFb was negatively associated with risk of secondary hypertension Similar trend was also found in MIP1b and MIG. Additionally, there was not any evidence of 41 inflammatory regulators associated with primary hypertension.

The TNF genes are located in the HLA complex on chromosome 6p21.3 and include TNF-α and TNFb, which play important roles in cell growth, differentiation, proliferation and immunity, apoptosis, and are involved in a wide range of disease progression. Tumor necrosis factor B (TNFB) is a cytokine produced by activated macrophages and is closely related to the TNFA gene (16). TNFB is a Th1 cytokine produced mainly by activated T and B lymphocytes. It is a potent mediator of inflammation and immune suppression (17). TNF-B was associated with serum triglyceride and very low density lipoprotein (VLDL) levels in interaction with TNF-B (18). The interaction between long-chain polyunsaturated fatty acids and nitric oxide, superoxide anions, and transforming growth factor beta can effectively prevent hypertension (19). Recently, it was found that inflammatory biomarkers were significantly elevated in a cohort of patients with idiopathic hypertension, with TNF-b and NT-proBNP being significantly correlated with 5-year survival (20). TNFB can induces an inflammatory response through activation of NF-kB nuclear proteins (16). TNFB promotes the progression of intestinal dermatitis in a YAP dependent manner (21). TGF *β* subtypes induce EndMT in human microvascular endothelial cells (22), indicating that TNF *β* is positively correlated with the progression of primary hypertension. However, in this Mendelian analysis, we found a negative correlation between TNF *β* and secondary hypertension. Previous studies have showed the cytokine TGF - *β* (transforming growth factor - *β*) is independent of the production of inflammatory cytokines and can increase the expression and activity of glycolytic enzyme PFKL (phosphofructokinase-1 liver type), promote glycolysis, but inhibit the production of pro-inflammatory cytokines (23). Dysregulation of BMP/TGF - *β* signaling is associated with disease progression in human PAH and rodent model PH (24). The integrated changes of TGF - *β* superfamily growth signals may lead to the pathogenesis of secondary hypertension (25). TGF - *β* (1) mRNA may be a useful gene therapy agent for treating hypertensive vascular diseases (26). Consistent with the findings of the present study, and TNF *β* may be a risk factors for primary hypertension, and a protect factor for secondary hypertension.

The macrophage inflammatory protein-1 (MIP-1) protein is structurally related to proinflammatory chemokine of the CC subfamily, one of the four chemokine groups defined by their primary structure (i.e., CXC, C, CC, and CX3C) (27). Macrophage Inflammatory Protein (MIP) 1b is a member of the inflammatory cytokine gene family whose expression is induced by pro-inflammatory and pro-mitotic stimuli (28).The gene is rapidly induced by LPS or IL-7 in human peripheral blood mononuclear cells, Elevated MIP1b gene is associated with an increased risk of total breast cancer and increased number of ER-positive BC cases (29). MIP-1β levels are elevated in the cerebrospinal fluid of patients with amyotrophic lateral sclerosis (30). Additionally, research has confirmed that MIP1b[ORIVW:0.92; 95% confidence interval: 0.85–0.98; *P* = 0.022% was negatively correlated with the risk of diabetes (31). Inhibition of macrophage factor MIP1b can mediate hypoxic pulmonary hypertension (32). No studies have reported a correlation between MIP1b and mortality or disease progression in patients with secondary hypertension, and the present study is the first to demonstrate a positive correlation between MIP1b and the progression of secondary hypertension.

Monokine induced by gamma interferon (MIG) induces adhesion of interleukin (IL)- 2 rapidly transiently processed T lymphocytes to immobilized integrin receptors through activation of the receptor CXCR3 on T cells (33). Previous studies have shown that the inflammatory chemokine MIG is increased in serum species of patients with left ventricular dysfunction (34). In serum of patients with idiopathic pulmonary arterial hypertension (IPAH) only IP-10 and MIG were significantly elevated (35). A model of 3 cytokines (IL-7, MIG, and SCF) was effective in predicting the multifactorial combination of PIH in ART pregnancies (36). MIG is an effective measure to improve compliance in hypertensive patients (15). MIG-7 and other MIG family molecules are positively correlated with the progression of hypertension (37, 38). While Mig-6 is necessary for proper lung development and adult lung homeostasis. MIG-6 negatively regulates STAT3 phosphorylation in uterine epithelial cells and inhibits inflammation progression (39). Consistent with the relationship between MIG and secondary hypertension obtained from the analysis of this study. Perhaps MIG-6 is negatively correlated with secondary hypertension.

The present study is the first to use MR in two samples to infer a causal relationship between inflammatory factors and blood pressure indices. Although recently published GWAS studies have helped to identify possible genetic causes of HP (40), there is the limitation of a small number of instrumental variables available. Estimates of the heritability of inflammation are variable depending on the method of calculation (41). However, it did succeed in identifying correlations between inflammation-related SNPs and systolic and diastolic blood pressure.

In conclusion, this study supported a negative relationship between TNFb, MIP1b 和 MIG6 and secondary hypertension. While the mechanism of this relationship warrants further study, we need to be confirmed in a large number of hypertensive patients. Also, the absence of a causal relationship found in the study between inflammation and primary hypertension requires future investigation (this could be due to the kind of inflammatory markers explored belong to CC chemokine family and had limited variety). This study suggested that hypertension can occur without traditional risk factors, so a shift in prevention methods is needed. Targeting secondary hypertension rather than just traditional risk factors is crucial for preventing adverse events. At the same time, personalized medicine and biomarker research can be combined to provide new ways to improve risk stratification, and integrate genetic and biomarker data, as well as artificial intelligence tools, which is expected to optimize hypertension risk management.

## Data Availability

Publicly available datasets were analyzed in this study. This data can be found here: https://r9.finngen.fi/pheno/I9_HYPTENSESS, https://r9.finngen.fi/pheno/I9_HYPTENSEC
